# Randomised, double-blind, placebo-controlled trial of oral probiotic *Streptococcus salivarius* M18 on head and neck cancer patients post-radiotherapy: a pilot study

**DOI:** 10.1038/s41598-020-70024-y

**Published:** 2020-08-06

**Authors:** Anna Vesty, Kim Gear, Sharon Boutell, Michael W. Taylor, Richard G. Douglas, Kristi Biswas

**Affiliations:** 1 Department of Surgery, The University of Auckland, Auckland, New Zealand; 2 Department of Otorhinolaryngology, Auckland District Health Board, Auckland, New Zealand; 3 Oral Health, Auckland University of Technology, Auckland, New Zealand; 4 School of Biological Sciences, The University of Auckland, Auckland, New Zealand; 5 Maurice Wilkins Centre for Molecular Biodiscovery, The University of Auckland, Auckland, New Zealand

**Keywords:** Head and neck cancer, Microbiome, Oral microbiology, Oral diseases

## Abstract

Xerostomia detrimentally affects the oral health of many head and neck cancer patients who undergo radiotherapy. Its sequelae become an ongoing burden for patients that often manifest as periodontal disease and dental decay. Bacteria play a major role in the pathogenesis of these conditions and here we explore the use of an oral probiotic to beneficially modulate the oral bacterial community post-radiotherapy. In this pilot study, a four-week intervention with oral probiotic lozenges containing *Streptococcus salivarius* M18 was trialled in seven patients. Post-intervention changes in oral health and in the composition of the plaque and saliva bacterial communities were compared with six patients in a placebo group. An improvement in periodontal screening and plaque index scores was observed in both groups after the intervention period. The oral probiotic lozenges did not significantly impact bacterial community composition or diversity, nor did the probiotic lozenges increase the relative sequence abundance of ZOTU_1 (the probiotic-associated sequence assigned to *S. salivarius*) detected in the samples. Network analyses suggest negative interactions occurred between ZOTU_1 and species from the periopathogenic genera *Campylobacter*, *Fretibacterium*, *Selenomonas* and *Treponema* but further investigation is required to more fully understand the beneficial properties of this oral probiotic.

## Introduction

Head and neck cancer describes a group of malignant tumours that often require radiotherapy as part of the treatment approach. Due to the close proximity of the salivary glands to the radiation portal, many head and neck cancer patients sustain permanent radiation-induced damage to the salivary glands during radiotherapy^1,2^. Subsequently, salivary gland function is reduced, resulting in chronic hyposalivation and xerostomia (dry mouth) that causes considerable discomfort, compromises mastication and speech, increases the risk of oral health complications and decreases the quality of life for many patients^1,3,4^.

Hyposalivation and loss of salivary buffering capacity lead to a decrease in the pH of saliva and a shift to acidogenic and cariogenic bacteria, including an increase the in abundance of *Streptococcus mutans* and *Lactobacillus* spp.^5–7^. These effects, combined with a decrease in the mechanical flushing mechanism of saliva to reduce plaque build-up, leave patients prone to post-radiotherapy gingivitis, periodontal disease and rampant dental decay^2,3,8^.

Oral probiotics, primarily those containing lactobacilli, have been successfully shown to decrease the abundance of periopathogens in sub- and supra-gingival plaque, significantly improve plaque index and periodontal pocketing scores and reduce periodontal inflammation^9–16^. Oral probiotic strain *S. salivarius* M18 has shown great promise owing to its ability to produce bacteriocin-like inhibitory substances (BLIS). In vitro, *S. salivarius* M18 has an inhibitory effect on the periodontal pathogens *Porphyromonas gingivalis* and *Prevotella intermedia*^17^ and decreases the expression of pro-inflammatory cytokines associated with periodontal disease, including interleukin (IL)-6 and IL-8^18^.

*S*. *mutans* is still considered the primary etiological agent of dental decay, although caries are increasingly characterised by their polymicrobial nature^19,20^. Previous reports show that the oral probiotic strain *S. salivarius* M18 can inhibit the growth of *S. mutans* and other mutans streptococci^21–23^. *S. mutans* establishes itself in the dental plaque biofilm where it ferments sucrose to produce lactic acid, which breaks down tooth enamel and leads to decay^8^. Oral probiotic lozenges containing *S. salivarius* M18 successfully decreased the incidence of new dental caries in a high risk group of children^24^.

The few studies that have investigated the impact of oral probiotics on the oral microbiota show that bacterial communities are not substantially altered by oral probiotic interventions^10,17,25^. However, the potential for oral probiotics to modulate the oral microbiota has not been applied to post-radiotherapy head and neck cancer patients, who are susceptible to oral health deterioration. Therefore, this pilot study of *S. salivarius* M18 attempts to characterise and correlate probiotic-induced changes in the oral bacterial community with the progression or stabilisation of periodontal disease and changes in dental plaque indices during a four-week intervention in post-radiotherapy head and neck cancer patients.

## Results

**Figure 1:**
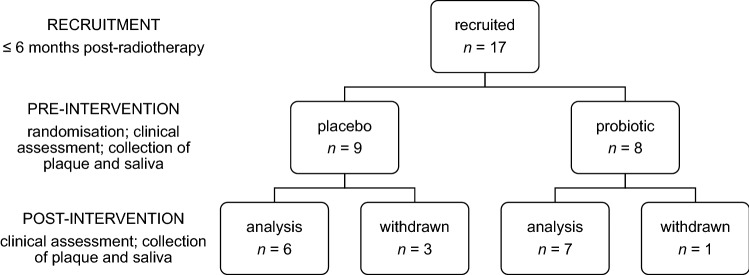
Study cohort.

This pilot study was conducted over a four-week intervention period with either oral probiotic lozenges containing *S. salivarius* M18 or placebo lozenges. Pre- and post-intervention oral health assessments were performed, coinciding with the collection of plaque and saliva samples for bacterial community analyses (Fig. 1).

### Study cohort

Seventeen patients were recruited for this pilot study; four were excluded post-randomisation for reasons beyond our control including: antibiotic treatment, failure to comply (lozenges not taken) and two patients were lost to follow-up (failed to attend) as described in Fig. 1. No significant pre-intervention differences were observed between the probiotic and placebo groups for the patient variables described in Table 1.

**Table 1.** Patient demographics.

Variable^a^	Placebo (*n* = 6)	Probiotic (*n* = 7)	*p* value
Gender, male:female	4:2	3:4	0.59
Age (years)	53.5 ± 17.1	53.3 ± 13.0	0.98
Total radiation dose (Gy)	65.5 ± 5.0	67.1 ± 4.5	0.56
Concomitant chemotherapy	3/6 (50%)	5/7 (71%)	0.59
Xerostomia (patient-reported)	5/6 (83%)	5/7 (71%)	1.00
Weeks post radiotherapy	11.0 ± 8.6	6.9 ± 2.5	0.21
Total lozenges taken	26 ± 3.0	27 ± 3.0	0.42
Pre-intervention CPITN	3.2 ± 0.9	2.7 ± 0.9	0.42
Pre-intervention O’Leary PI	48 ± 30%	37 ± 25%	0.54

*CPITN* community periodontal index of treatment needs, *PI* plaque index.

^a^Categorical variables are reported as proportion yes/total (%), with the exception of gender, which is given as male:female ratio, no. (%). Continuous variables are summarised as mean ± standard deviation.

### Comparison of pre- and post-intervention clinical measures of oral health

After the four-week intervention, an improvement in Community Periodontal Index of Treatment Needs (CPITN)^26^ scores was observed in three patients: 2/6 in the placebo group and 1/7 in the probiotic group, but the difference between the two groups was not significant (*p* > 0.05). The CPITN scores for all other patients remained stable. Post-intervention, an improvement in O’Leary Plaque Index^27^ scores was observed in both groups, with the average percentage of tooth surfaces with plaque falling (albeit not significantly) from 37 to 26% in the probiotic group and from 48 to 32% in the placebo group. No significant reduction in tooth surfaces with plaque was observed between the probiotic and placebo groups (*p* > 0.05). O’Leary Plaque Index scores were not available for one patient in the probiotic group.

### Probiotic viability

No growth was observed from the placebo lozenges; > 1 × 10^9^ CFU/L of growth resembling alpha haemolytic streptococci was recovered from the culture of a BLIS M18 lozenge obtained from a sealed study container, and a representative colony was identified as belonging to the *S. salivarius* group.

### Bacterial community profiles

Post filtering and processing of the 52 patient samples, 1,117,173 bacterial 16S rRNA gene sequences were obtained and classified into 1,247 zero-radius operational taxonomic units (ZOTUs). Rarefaction to 3639 sequences per sample was sufficient to capture the vast majority of bacterial diversity (see Supplementary Fig. S1 online) and resulted in 1102 ZOTUs classified into 117 genera in plaque samples and 931 ZOTUs classified into 106 genera in saliva samples. Of the 27,863 bacterial sequences obtained from the BLIS M18 probiotic lozenge, 99.8% clustered with ZOTU1, which was assigned to *S. salivarius*.

**Figure 2:**
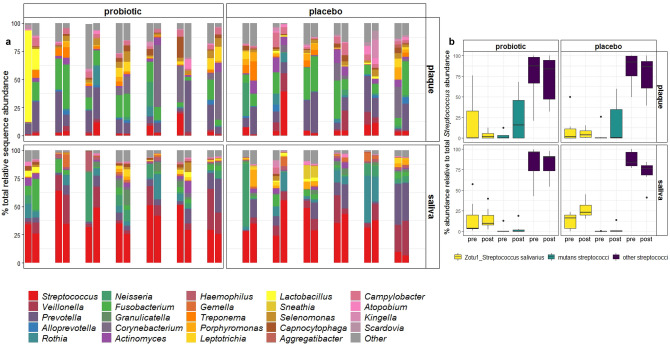
(**a**) Genus-level summary of bacterial communities present in plaque and saliva. Each pair of bars represents one patient, with pre-intervention sample on the left and post-intervention sample on the right of the pair. Plaque and saliva samples are vertically aligned by corresponding patient; (**b**) boxplots expressing relative sequence abundance of ZOTU1_*S*. *salivarius*, mutans streptococci and all other streptococci, relative to the total relative sequence abundance of all *Streptococcus* sequences. The solid line within each box indicates the median value for each group; data were compared using ANOVA with Tukey’s post-hoc test.

The plaque microbiota was dominated by the anaerobic Gram-negative genera *Prevotella* and *Fusobacterium*, which comprised an average of 17% and 15% of the plaque bacterial community, respectively. The Gram-positive genus *Corynebacterium* was the next most abundant at 8% on average, however its presence varied between patients and was absent altogether in some plaque samples (Fig. 2a). *Streptococcus* followed at an average of 6% of the plaque bacterial community. In saliva samples, *Streptococcus* was the most abundant genus, accounting for 35% of the bacterial community, on average, across all samples. *Veillonella* and *Prevotella* were the next most abundant genera, comprising over 10% of the salivary microbiota at 12% and 10% on average, respectively (Fig. 2a).

### Comparison of *Streptococcus* ZOTUs pre- and post-intervention

The total average relative sequence abundance of *Streptococcus* was significantly higher in saliva samples at 35 ± 2.6% (mean ± SE), compared to an average of 6 ± 1.6% in plaque samples (*p* < 0.001). In the probiotic group, the average relative abundance of *Streptococcus* decreased slightly post-intervention in both plaque and saliva samples, although this decrease was not significant (*p* > 0.05). Conversely, the average relative abundance of *Streptococcus* increased in the placebo group for both plaque and saliva samples but not significantly (*p* > 0.05).

The abundance of ZOTU1_*S. salivarius*, which was associated with the BLIS M18 lozenges in this study, was examined relative to the total abundance of all *Streptococcus* ZOTUs for each sample both pre- and post-intervention, alongside changes in the abundance of ZOTUs assigned to mutans streptococci. The three other ZOTUs that were assigned to *S. salivarius* were included in the ‘other streptococci’ group (see Supplementary Table S2 online). ZOTUs assigned to mitis group streptococci (*S. oralis*, *S. sanguinis*, *S. parasanguinis*, *S. gordonii* and *S. cristatus*) and *S. intermedius* (anginosus group) were found in the highest abundances in ‘other streptococci’, on average, in most samples (Supplementary Table S2 online).

The average abundance of ZOTU1_*S. salivarius* relative to total *Streptococcus* abundance was higher in saliva samples at 17 ± 3.0%, compared to 10 ± 3.7% in plaque, while the average abundance of mutans streptococci relative to total *Streptococcus* abundance was higher in plaque at 13 ± 4.2% compared to 2 ± 1.0% in saliva. The differences for each species between specimens was not significant (*p* > 0.05), and the majority of the *Streptococcus* abundance was grouped as ‘other streptococci’ for both plaque (77 ± 4.9%) and saliva (81 ± 3.3%). Overall, no significant changes in the average abundances of ZOTU1_*S*. *salivarius* or mutans streptococci (relative to total *Streptococcus* abundance) were observed pre- and post-intervention, within specimen type (*p* > 0.05, Fig. 2b).

### Bacterial community diversity pre- and post-intervention

Alpha diversity, represented by the number of ‘observed species’ present, remained stable pre- and post-intervention for both groups. Overall, the number of observed species was the same for the combined plaque samples and the combined saliva samples, both with an average of 182 ± 12 observed species (*p* > 0.05). No significant changes in alpha diversity were observed in the plaque samples, with diversity falling slightly in the probiotic group from 176 ± 31 observed species pre-intervention to 172 ± 21 post-intervention, while rising from 186 ± 17 observed species pre-intervention to 196 ± 26 post-intervention in the placebo group (*p* > 0.05). Alpha diversity increased slightly post-intervention in saliva samples from both groups, from 177 ± 30 pre-intervention to 205 ± 25 observed species post-intervention in the probiotic group and from 165 ± 20 to 179 ± 23 observed species in the placebo group, however the pre- and post-intervention differences within or between intervention groups were not significant (*p* > 0.05).

**Figure 3:**
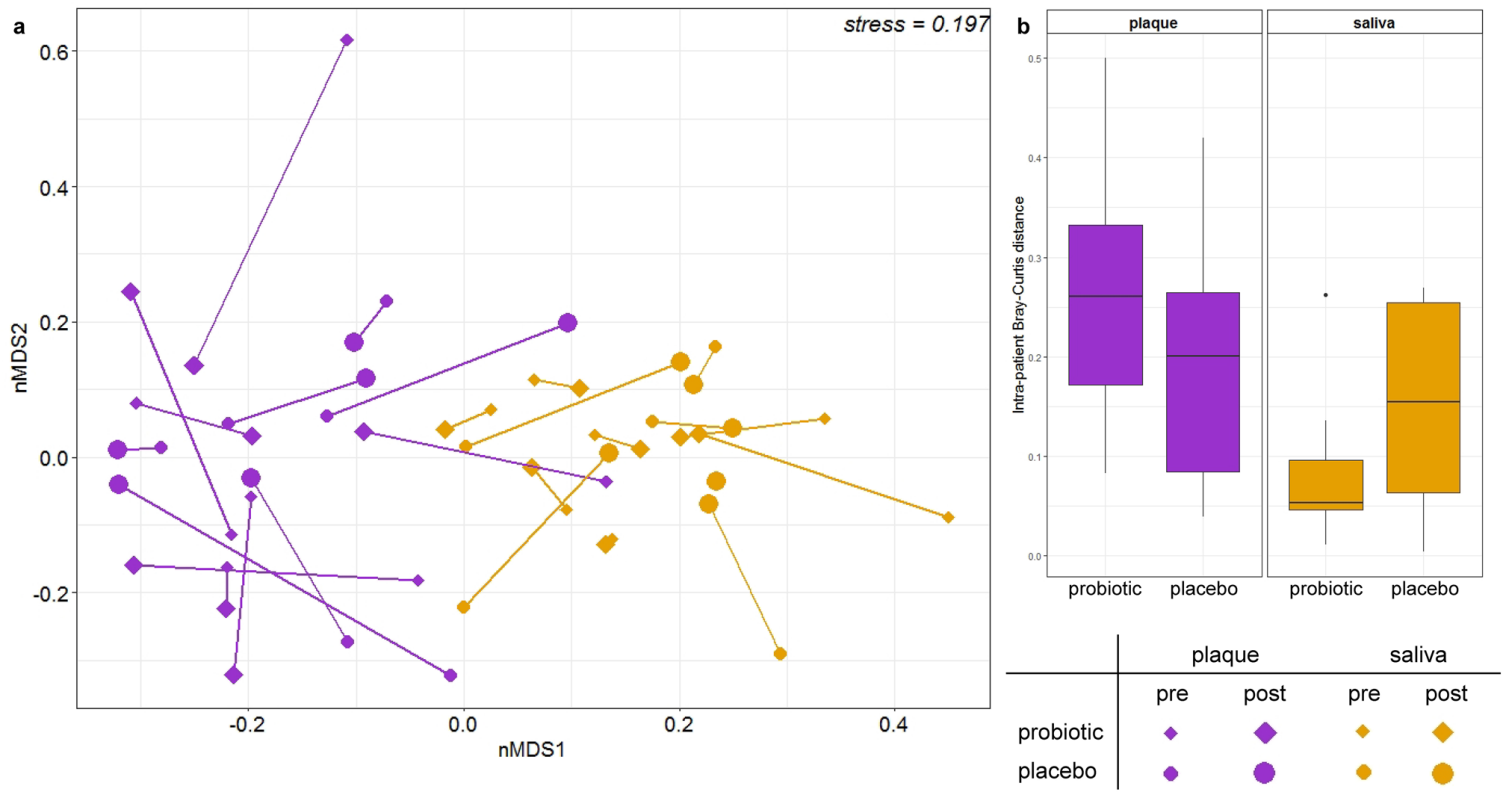
(**a**) nMDS plot based on Bray–Curtis dissimilarity of all samples. Each point indicates one sample (according to the legend) and pairs of pre- and post-intervention samples for each patient are joined by vector lines, within sample type; (**b**) boxplot of intra-patient Bray–Curtis distance pre- and post-intervention by intervention group for plaque and saliva. The solid line within each box indicates the median value for each group; data were compared using a two-way ANOVA.

Visualisation of Bray–Curtis dissimilarity through nMDS revealed that samples significantly clustered by specimen type, accounting for 15% of variation in the model (*p* < 0.001, Fig. 3a). Intra-patient Bray–Curtis distances between pre- and post-intervention plaque samples from both groups were greater than the distances observed between pre- and post-intervention saliva samples from both groups, suggesting salivary microbiota were more stable in this study than the plaque microbiota (*p* < 0.05). No significant differences in pre- and post-intervention Bray–Curtis distance were observed within sample type between the probiotic and placebo groups (*p* > 0.05, Fig. 3b).

### Inference of bacterial community networks in plaque and saliva

**Figure 4:**
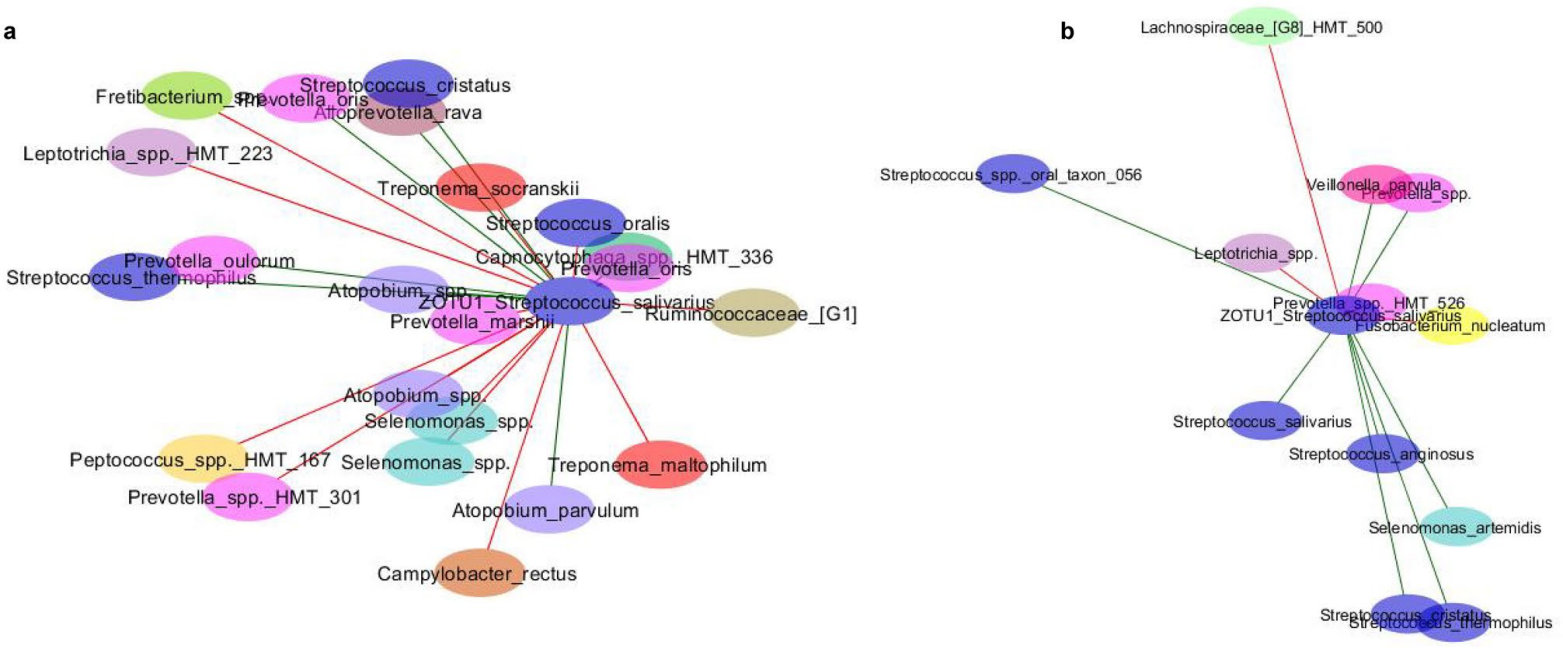
Selected nodes from the plaque bacterial network in (**a**) and the salivary bacterial network in (**b**) based on interactions with ZOTU1_*S*. *salivarius*. Green and red edges represent positive and negative interactions, respectively; nodes are coloured by genus. HMT, human microbial taxon.

The bacterial community network inferred from all plaque samples contained 8591 edges, of which 6450 were positive interactions and 2141 negative. ZOTU1_*S*. *salivarius*, associated with the probiotic lozenges, had a closeness centrality of 0.38 and its clustering coefficient was 0.04. In the plaque network, ZOTU1_ *S*. *salivarius* contained 22 direct edges and nine of these edges represented positive interactions, while the remaining 13 were negative interactions (Fig. 4a). Negative interactions occurred between ZOTU1_ *S*. *salivarius* and ZOTUs assigned to periopathogenic taxa including *Campylobacter rectus*, *Fretibacterium* spp., *Treponema maltophilum*, *T. socranskii* and *Selenomonas* spp. (Fig. 4a).

The combined saliva samples generated a lower density network than the plaque samples that contained 5070 edges, 3788 of which were positive interactions and 1282 negative. In the saliva network, ZOTU1_ *S*. *salivarius* had a closeness centrality of 0.35 and a clustering coefficient of 0.06. ZOTU1_ *S*. *salivarius* had fewer edges in the saliva network, forming 13 direct edges. Eight of these edges represented positive interactions (Fig. 4b), five of which were with other ZOTUs assigned to the *Streptococcus* genus, including ZOTU897 also assigned to *S. salivarius*. Inverse covariances for the interactions in Fig. 4 are described in Supplementary Table S3.

## Discussion

In this pilot study, a four-week intervention with *S. salivarius* M18 in the form of an oral probiotic lozenge had minimal impact on the composition and diversity of the plaque and salivary microbiota in post-radiotherapy head and neck cancer patients. These findings are consistent with previous trials that show probiotic streptococci do not effectively increase the overall abundance of oral streptococci and that the temporal persistence of probiotic streptococci is limited^17,25^. *Streptococcus* forms part of the core resident oral microbiota^28,29^, making it difficult to detect meaningful changes in the relative abundance of this resident genus. Furthermore, detecting *S. salivarius* M18 and its effect on the oral microbiota requires strain-level analysis, attempted in this study using ZOTUs as a proxy for strain-level identification. Culture-based analyses have an advantage over sequencing-based studies in this context in that BLIS production can be detected phenotypically, thereby gaining a more accurate enumeration of *S. salivarius* M18 carriage. Such methods have demonstrated that the persistence of *S. salivarius* M18 is dose-dependent^17^. Strain-specific Droplet Digital PCR or real-time qPCR could be considered as alternative molecular approaches to measure total abundance, rather than relative abundance. For example, the TaqMan-based quantitative PCR screening assay developed for the probiotic strain *S. salivarius* K12 to detect colonisation efficacy^30^. Such approaches are also applicable to quantifying changes in the total abundance of pathogenic species, including *S. mutans*.

Core salivary bacterial genera, including *Streptococcus*, remain stable during radiotherapy^31^. However, subsequent hyposalivation and xerostomia, reported by 10 of the 13 patients in this study, may compromise the ability of probiotic strains to disseminate and colonise the oral cavity. Reduced susceptibility to probiotic-induced changes in the oral microbiota may result from radiotherapy-induced changes in the pH, volume and viscosity of saliva. These changes might increase the microbial concentration of saliva, limiting its capacity to further increase its microbial load, and could also affect the expression of bacteriocins and adhesion factors. We speculate that the lack of significant increase in the relative sequence abundance of ZOTU1_*S. salivarius* in the probiotic group post-intervention could be attributable to these mechanisms. Conducting a similar trial on a healthy cohort may provide further insight into the capacity of *S. salivarius* M18 to alter oral bacterial communities. Our data also suggest that the probiotic was not able to penetrate the plaque biofilm, as there was no significant increase in the relative sequence abundance of ZOTU1_*S. salivarius* in the post-intervention plaque samples from the probiotic group. Even so, a lack of significant changes in the oral microbiota may in fact support the safety of the probiotic, as a major disturbance could have detrimental effects.

While this pilot study failed to find any significant abundance-based changes in *S. salivarius* or decay-associated mutans streptococci, other microbial mechanisms that affect post-radiotherapy oral health may be relevant. Based on bacterial network analyses, there may be motive to consider the use of BLIS M18 clinically. In the saliva network, ZOTU1_*S. salivarius* positively interacted with other core microbiota, including *Prevotella*, *Veillonella* and several *Streptococcus* species but negatively interacted with *Fusobacterium nucleatum*, supporting previous observations that *Streptococcus* and *Fusobacterium* are negatively correlated in saliva^32^. In the plaque network, several negative interactions were detected between ZOTU1_*S*. *salivarius* and species from the periopathogenic genera *Campylobacter*, *Fretibacterium*, *Selenomonas* and *Treponema*. However, the data presented here is based on a pilot study and the long-term benefit of these interactions should be investigated in additional trials that include larger cohorts and a longer intervention period. Such studies may benefit from investigating the effect of *S. salivarius* M18 on salivary pH and the incidence of dental caries, and could gain valuable insight into how probiotic intervention impacts quality of life by evaluating physical and psychological measures specific to this cohort of patients^33^.

An overall improvement in the O’Leary Plaque Index score was seen in both intervention groups in this study, suggesting clinical improvements were not driven by probiotic-induced changes in the oral microbiota. We speculate that the oral hygienist involvement may have contributed to this improvement by providing oral hygiene advice that was given in order to standardise the oral health regime for all participants in the trial. This advice may have resulted in the improvement of brushing and/or inter-dental cleaning routines for some patients compared to their pre-trial habits.

The use of oral probiotics to modulate host immune responses and microbial interactions through antagonism and co-aggregation is a promising mechanism to improve oral health^34^. However, these beneficial properties require further optimisation and exploitation before oral probiotics are clinically recommended as a complementary approach to improve post-radiotherapy oral health for head and neck cancer patients.

## Methods

### Patient enrolment and randomisation

Seventeen patients were enrolled at Auckland City Hospital, Auckland, New Zealand for this randomised, double-blinded, placebo-controlled study of the oral probiotic BLIS M18 (BLIS Technologies Ltd, Dunedin, New Zealand). This study was conducted on the selected patient cohort with approval granted by the national Health and Disability Ethics Committee (16/STH/123) and the Auckland District Health Board (7396). All participants provided written informed consent. The trial was registered with the Australian New Zealand Clinical Trials Registry on the 17/05/2017 (Registration # ACTRN12617000710325) and carried out in accordance with relevant guidelines and regulations. Recruitment for this study began in October 2017 and follow-up was completed in November 2018.

Non-palliative, fully or partially dentate patients who had received ≥ 60 Gy of radiation to the head and neck region in the previous six months were eligible for inclusion. Patients requiring antibiotic treatment were excluded. Patients generally reported a low carbohydrate diet and did not require percutaneous endoscopic gastrostomy feeding. Patients were randomly allocated into Group A (placebo) or Group B (probiotic) by block randomisation with a 1:1 ratio. As a pilot study, the effect size was not calculated but the data generated were used to investigate the feasibility of a larger study. Patients, the dental hygienist assessing oral health and the researcher responsible for randomisation were masked to the treatment/placebo allocation of the lozenges. Certificates of analysis with microbial specifications for each of the Group A and Group B lozenges were provided by the manufacturers and remained undisclosed during the trial period.

### Study design and sample collection

Pre-intervention, unstimulated saliva samples were collected in a sterile container; 1 mL of saliva was requested but patients usually provided less due to hyposalivation. Sub- and supra-gingival plaque was collected pre-intervention from the posterior mandibular molars and, if insufficient plaque was available, the lower incisors. Pre-intervention oral health was assessed as detailed below. Patients were given 30 of the randomly assigned Group A or B lozenges: Group A lozenges were a placebo; Group B lozenges contained 3.5 × 10^9^ CFU of *S. salivarius* M18 (BLIS M18) per lozenge at the time of manufacture (BLIS Technologies Ltd, Dunedin, New Zealand). Patients were asked to suck one lozenge slowly until dissolved after brushing in the evening every day for four weeks. Oral hygiene advice during the trial complemented recommendations for post-radiotherapy oral care. Patients were advised to brush twice daily using high fluoride toothpaste, with no post-brushing rinse and to clean interdentally. Four weeks post-intervention, plaque and saliva samples were collected and post-intervention oral health assessments were performed, as described above. Plaque samples, collected in RNA*later* (Life Technologies, Auckland, New Zealand), and neat saliva samples were stored at − 20 °C until further analysis.

### Oral health assessments

CPITN scores were used to screen for gingival bleeding, calculus and periodontal pocketing^26^. Dentition was divided into six sextants, and each sextant was scored between 1 and 4, with the highest score reported. A score of 0 indicates no periodontal disease. Scores 1–4 respectively reflect increasing treatment needs: 1, gingival bleeding on probing, 2, calculus,3, pocketing 4–5 mm; 4, pocketing ≥ 6 mm. Scores of 3 and 4 indicate periodontal disease. The O’Leary Plaque Index was used to quantify plaque on tooth surfaces^27^. Each tooth was divided into four surfaces (buccal, distal, lingual and mesial), and the presence of plaque on each surface was recorded for all teeth present. The number of plaque-containing surfaces was divided by the total number of tooth surfaces available and reported as a percentage for each patient. All oral health assessments were performed by the same qualified oral hygienist.

### Assessment of probiotic viability

After trial completion, one lozenge from each group was separately dissolved in 10 mL of sterile water. One μL of each suspension was inoculated on tryptic soy agar with sheep blood and incubated at 35 °C with 5% CO_2_ for 32 h. Colonies were enumerated and a representative colony was identified by MALDI-TOF mass spectrometry using the VITEK MS system (bioMérieux, Marcy l'Etoile, France).

### DNA extraction and sequencing preparation

One lozenge from each of groups A and B was dissolved separately in 1 mL of sterile, PCR-grade water. Aliquots of the clinical samples and the dissolved lozenges were mechanically lysed using Lysing Matrix E bead tubes and RLT Plus lysis buffer for 30 s × 2 using the Omni Bead Ruptor 24. Purification of genomic DNA was achieved with the AllPrep DNA/RNA Isolation Kit (Qiagen, NRW, Germany), using a spin column for DNA isolation. PCR-grade water was used as a negative extraction control.

The V3-V4 region of the bacterial 16S rRNA gene was amplified using Illumina-compatible primers S-D-Bact-0341-b-S-17 and S-D-Bact-0785-a-A-21^35^^,^ as previously described^32^. Duplicate PCR reactions were pooled and purified for sequencing, as previously described^29^. Normalised samples were submitted to Auckland Genomics Ltd for sequencing on the Illumina MiSeq platform.

### Bioinformatic analyses

Bacterial 16S rRNA gene sequence data from plaque and saliva samples were processed in USEARCH (v10) using the UNOISE algorithm to cluster sequences into ZOTUs^36,37^. This approach enabled better resolution than OTU-based methods and was applied to closely examine *Streptococcus* species in the data^38^. In particular, the relative sequence abundances of *S. salivarius* (ZOTU1) and mutans streptococci were compared by intervention group, within sample type.

Primer-binding regions were trimmed before sequences were quality filtered and merged to remove sequences with a minimum merge length of < 350 bp and > 5 mismatches. Filtered sequences were clustered into ZOTUs using the ‘-unoise3′ command, with a minimum cluster size of 8^36^. ZOTUs were mapped to the filtered sequence data with a minimum identity threshold of 99% in order to create a ZOTU table. Taxonomic prediction was performed using the RDP classifier^39^ against the Human Oral Microbiome Database (v15.1)^40^. ZOTUs observed in < 3 samples were filtered from the final ZOTU table. *Streptococcus* ZOTUs unresolved to species-level were further classified using BLASTn, with species-level taxonomic classification based on ≥ 98% sequence similarity (see Supplementary Table  S2 online). Alpha and beta diversity measurements were estimated in QIIME (v1.9)^41^.

### Network analyses

Microbial networks in plaque and saliva were inferred independently using SPIEC-EASI (SParse InversE Covariance Estimation for Ecological Association Inference), implemented using the Spiec-Easi R package^42^. Unrarefied ZOTU tables were filtered and normalised to retain only ZOTUs with a minimum occurrence in 10% of samples. The SPIEC-EASI sparse neighbourhood and inverse covariance selection algorithms were used for data transformation and estimation of the interaction graph, based on Meinshausen-Buhlmann’s neighbourhood selection method^42^. The network was visualised in Cytoscape (v3.4.0)^43^^,^ and nodes and edges that interacted with ZOTU1_*S. salivarius* were selected out. Inverse covariance was used to visually map the positive and negative edges of the selected network.

### Statistical analyses

Patient demographics were compared between the probiotic and placebo groups using Fisher’s exact test for categorical variables and a one-way analysis of variance (ANOVA) for continuous variables, α = 0.05. Differences in pre- and post-intervention CPITN scores for each patient were used to categorise clinical responses as either an improvement (difference < 0) or no improvement (≥ 0) observed. Differences were calculated by subtracting the pre-intervention CPITN score from the post-intervention score. Fisher’s exact test was used to statistically compare the clinical response categories between the probiotic and placebo groups. A two-way ANOVA with Kenward-Roger’s approximation was conducted and visualised in R (v3.6.0) to compare O’Leary Plaque Index scores by time point (pre- and post-intervention), intervention group and individual. Bray–Curtis dissimilarity was used to calculate intra-patient variability pre- and post- intervention by sample type and was visualised through non-metric multidimensional scaling (nMDS), with differences in Bray–Curtis distance statistically compared by intervention group using two-way ANOVA in R (v3.6.0)^44^. Partitioning of the distance matrix by variable was assessed using the *Adonis* function. Changes in alpha diversity pre- and post-intervention and the abundance of *Streptococcus* ZOTUs were visualised and statistically assessed by ANOVA with Tukey’s post-hoc test for multiple comparison of means in R (v3.6.0)^44^.

## Supplementary information

Supplementary Figure S1.

Supplementary Table S2.

Supplementary Table S3.

## Data Availability

The datasets analysed during the current study are available in the NCBI Sequence Read Archive (https://www.ncbi.nlm.nih.gov/sra) under Accession Number PRJNA588128.
