# Supplementary material for: Randomised, double-blind, placebo-controlled trial of oral probiotic Streptococcus salivarius M18 on head and neck cancer patients post-radiotherapy: a pilot study
Source: Sci Rep. 2020 Aug 6;10:13201. doi: 10.1038/s41598-020-70024-y (PMC7411050; doi:10.1038/s41598-020-70024-y)
Supplement: Supplementary file 1 — Supplementary Figure S1. [file 41598_2020_70024_MOESM1_ESM.pdf]

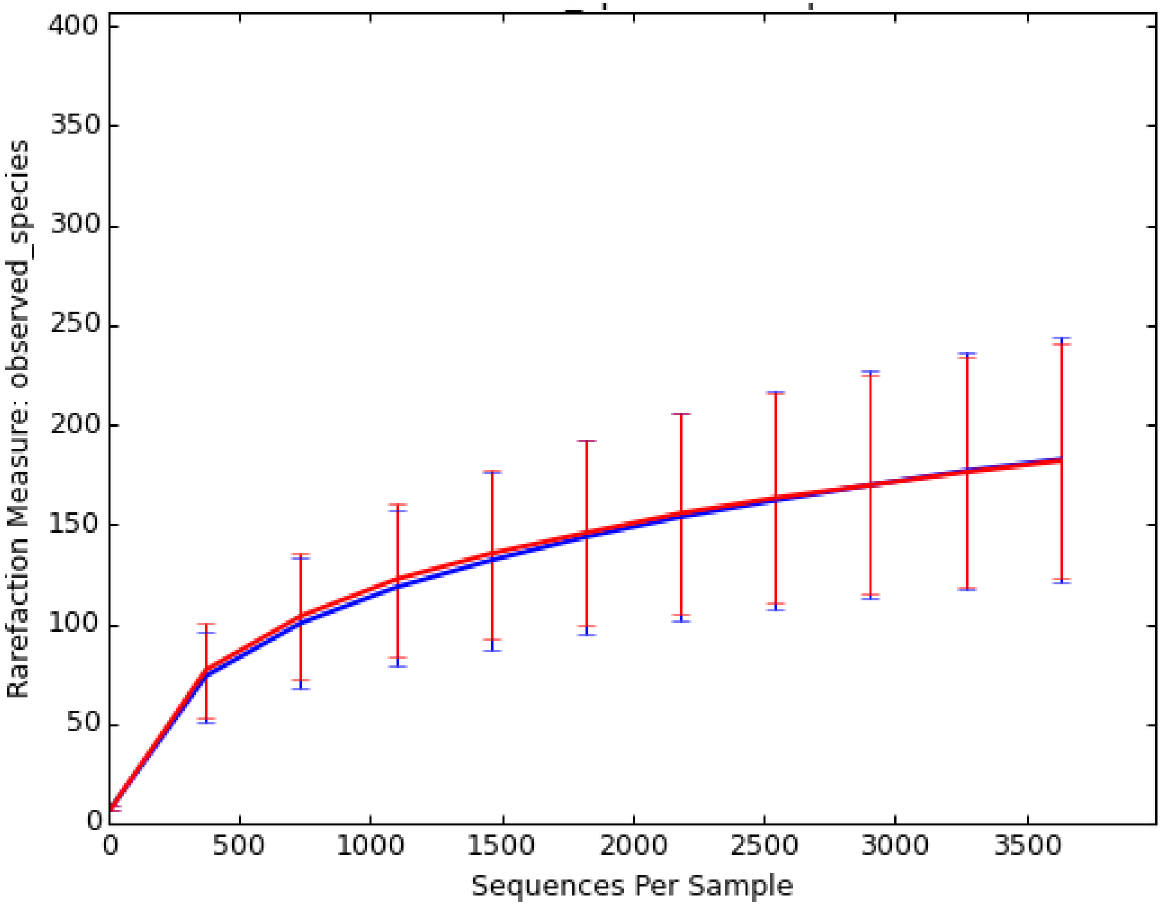

Supplementary Figure S1. Alpha diversity rarefaction analysis based on the ‘observed species’ metric by sample type as a function of the number of sequences per sample. Average for plaque samples in red and saliva samples in blue.
