# Supplementary material for: Randomised, double-blind, placebo-controlled trial of oral probiotic Streptococcus salivarius M18 on head and neck cancer patients post-radiotherapy: a pilot study
Source: Sci Rep. 2020 Aug 6;10:13201. doi: 10.1038/s41598-020-70024-y (PMC7411050; doi:10.1038/s41598-020-70024-y)
Supplement: Supplementary file 2 — Supplementary Table S2. [file 41598_2020_70024_MOESM2_ESM.pdf]

Supplementary Table S2. *Streptococcus* ZOTUs

(a) *Streptococcus* ZOTUs in plaque samples from probiotic cohort

**ZOTU classification (HOMD v15.1)**	**BLAST[1]**	**2a[2]**	**2b**	**4a**	**4b**	**6a**	**6b**	**8a**	**8b**	**10a**	**10b**	**12a**	**12b**	**14a**	**14b**	**16a**	**16b**
**Zotu1_*Streptococcus salivarius***	n/a	22	9	15	0	0	0	30	38	0	1	0	1	0	0	0	0
**Zotu2_*Streptococcus oralis_subsp._tigurinus_clade*_071**	n/a	2	6	27	60	0	0	0	255	38	9	5	21	493	1	25	7
**Zotu22_*Streptococcus parasanguinis_clade*_411**	n/a	1	6	0	6	0	0	15	32	0	0	157	1	0	0	0	1
**Zotu26_*Streptococcus mutans***	n/a	1	24	0	0	0	0	0	0	0	0	0	44	17	15	14	4
**Zotu35_*Streptococcus salivarius***	n/a	0	0	0	0	0	0	0	0	0	0	0	0	0	0	0	0
**Zotu37_*Streptococcus* species**	*Streptococcus gordonii*	0	0	1	11	0	0	0	68	8	11	0	12	154	0	40	9
**Zotu44_*Streptococcus species***	*Streptococcus sanguinis*	0	0	0	0	0	0	0	11	8	23	5	0	1	0	0	0

**Zotu46_*Streptococcus intermedius***	n/a	2	4	22	69	8	68	6	10	1	0	4	1	3	1	32	0
**Zotu59_*Streptococcus oralis_subsp._tigurinus_clade_071***	n/a	0	0	0	0	0	0	0	1	0	0	90	0	3	0	0	0
**Zotu64_*Streptococcus* species**	*Streptococcus parasanguinis*	0	6	0	0	0	0	0	5	0	0	6	0	3	0	0	0
**Zotu93_*Streptococcus anginosus***	n/a	0	0	0	0	0	7	10	0	1	0	0	0	0	0	1	0
**Zotu97_*Streptococcus constellatus***	n/a	0	8	9	0	93	22	0	0	1	2	0	0	1	5	0	0
**Zotu133_*Streptococcus* species**	*Streptococcus cristatus*	0	1	0	0	0	0	0	0	0	3	0	0	11	0	0	2
**Zotu168_*Streptococcus anginosus***	n/a	0	0	0	1	0	0	0	0	0	0	0	0	0	0	1	0
**Zotu180_*Streptococcus* species**	*Streptococcus* species	0	0	0	0	0	0	0	0	0	0	1	0	0	0	0	0
**Zotu201_*Streptococcus* species**	*Streptococcus peroris/lactarius*	0	0	0	0	0	0	0	0	0	0	32	0	0	0	0	0
**Zotu267_*Streptococcus* species**	*Streptococcus cristatus*	0	0	0	0	0	0	0	0	0	0	0	0	0	0	0	0
**Zotu299_*Streptococcus oralis_subsp._tigurinus_clade_071***	n/a	0	1	4	0	0	0	0	0	0	0	1	0	1	0	0	0

**Zotu335_*Streptococcus* species**	*Streptococcus anginosus*	0	0	3	0	0	0	0	0	0	0	0	0	0	0	0	0
**Zotu340_*Streptococcus oralis_subsp._tigurinus_clade*_071**	n/a	0	0	0	0	0	0	0	0	0	0	4	0	0	0	0	0
**Zotu341_*Streptococcus* species**	*Streptococcus* species oral taxon 056	0	0	1	0	0	0	0	0	0	0	0	0	0	0	0	0
**Zotu355_*Streptococcus* species**	*Streptococcus salivarius*	0	0	0	0	0	0	0	0	0	0	0	0	0	0	0	0
**Zotu517_*Streptococcus anginosus***	n/a	0	0	0	0	0	0	0	0	0	0	0	0	0	0	0	0
**Zotu615_*Streptococcus* species**	*Streptococcus* species	0	0	1	0	0	0	0	0	0	0	0	0	0	0	0	0
**Zotu668_*Streptococcus sobrinus***	n/a	1	4	0	0	0	0	0	0	0	0	0	0	0	0	0	0
**Zotu774_*Streptococcus parasanguinis_clade*_411**	n/a	0	0	0	0	0	0	0	0	0	0	0	0	0	0	0	0
**Zotu779_*Streptococcus* species**	*Streptococcus oralis*	0	0	0	0	0	0	0	0	0	0	0	0	0	0	0	0
**Zotu820_*Streptococcus* species**	*Streptococcus anginosus* group	0	0	0	0	0	0	0	0	0	0	0	0	0	0	0	0
**Zotu882_*Streptococcus* species**	*Streptococcus* species	0	0	0	0	0	0	0	0	0	0	0	0	0	0	0	0

**Zotu897_*Streptococcus* species**	*Streptococcus salivarius*	0	0	0	0	0	0	0	0	0	0	0	0	0	0	0	0
**Zotu933_*Streptococcus* species**	*Streptococcus* species	0	0	0	0	0	0	0	0	0	0	0	0	0	0	0	0
**Zotu1039_*Streptococcus* species**	*Streptococcus cristatus*	0	0	1	0	0	0	0	0	0	0	0	0	0	0	0	0
**Zotu1085_*Streptococcus* species**	*Streptococcus thermophilus*	0	1	0	0	0	0	0	0	0	0	0	0	0	0	0	0
**Zotu1130_*Streptococcus* species**	*Streptococcus pneumoniae*	0	0	0	0	0	0	0	0	0	0	0	0	0	0	0	0
**Zotu1174_*Streptococcus* species**	*Streptococcus anginosus*	0	0	0	0	0	0	0	0	0	0	0	0	0	0	0	0
**Zotu1200_*Streptococcus* species**	*Streptococcus gordonii*	0	0	0	0	0	0	0	1	0	0	0	0	0	0	0	0
**Zotu1214_*Streptococcus mutans***	n/a	0	1	0	0	0	0	0	0	0	0	0	1	6	0	0	0
**Zotu1218_*Streptococcus* species**	*Streptococcus* species	0	0	0	0	0	0	0	0	0	0	0	0	0	0	0	0
**Zotu1243_*Streptococcus* species**	*Streptococcus cristatus*	0	0	3	0	0	0	0	1	0	0	0	0	2	0	0	0

**Zotu1252_*Streptococcus mutans***	n/a	0	1	0	0	0	0	0	0	0	0	0	0	0	0	0	0
**Zotu1290_*Streptococcus* species**	*Streptococcus* species	0	0	0	0	0	0	0	0	0	0	0	0	2	0	0	0
**Zotu1300_*Streptococcus parasanguinis_clade_*411**	n/a	0	0	0	0	0	0	0	0	0	0	0	0	0	0	0	0
**Zotu1309_*Streptococcus* species**	no match/low cover	0	0	0	0	0	0	0	0	0	0	0	0	0	0	0	0
**Zotu1329_*Streptococcus* species**	*Streptococcus* species	0	0	0	0	0	0	0	0	0	0	0	0	0	0	0	0
**Zotu1346_*Streptococcus* species**	*Streptococcus oralis*	0	0	2	0	0	0	0	0	0	0	0	0	0	0	0	0
**Zotu1348_*Streptococcus* species**	no match/low cover	0	0	0	0	0	0	0	1	0	0	2	0	1	0	0	0
**Zotu1412_*Streptococcus* species**	no match/low cover	0	0	0	0	0	0	0	0	0	0	1	0	0	0	0	0
**Zotu1415_*Streptococcus* species**	*Streptococcus* species	0	0	0	0	0	0	0	0	0	0	0	0	1	0	0	0
**Zotu1427_*Streptococcus* species**	no match/low cover	0	0	0	0	0	0	0	0	1	0	0	0	14	0	0	2
**Zotu1430_*Streptococcus* species**	*Streptococcus parasanguinis*	0	0	0	0	0	0	0	0	0	0	0	0	0	0	0	0
**Zotu1431_*Streptococcus* species**	*Streptococcus cristatus*	0	0	0	0	0	0	0	0	0	0	0	0	0	0	0	0

**Zotu1444_*Streptococcus* species**	*Streptococcus gordonii*	0	0	0	0	0	0	0	0	0	0	0	0	0	0	0	0
**Zotu1466_*Streptococcus* species**	*Streptococcus gordonii*	0	0	0	0	0	0	0	0	0	0	0	0	0	0	0	0

HOMD, Human Oral Microbiome Database (v15.1); Zotu, zero-radius OTU

[1]BLAST classification based on ≥ 98% sequence similarity

[2]a, pre-intervention; b, post-intervention

(b) *Streptococcus* ZOTUs in plaque samples from placebo cohort

**ZOTU classification (HOMD v15.1)**	**BLAST[1]**	**3a**	**3b**	**7a**	**7b**	**9a**	**9b**	**13a**	**13b**	**15a**	**15b**	**17a**	**17b**
**Zotu1_*Streptococcus salivarius***	n/a	0	3	20	14	0	4	3	2	181	63	1	0
**Zotu2_*Streptococcus oralis_subsp._tigurinus_clade*_071**	n/a	77	6	20	68	70	37	9	42	2	4	79	115
**Zotu22_*Streptococcus parasanguinis_clade*_411**	n/a	0	0	0	262	0	0	0	0	34	11	1	0
**Zotu26_*Streptococcus mutans***	n/a	0	0	0	812	0	0	40	71	1	4	1	0
**Zotu35_*Streptococcus salivarius***	n/a	0	0	0	0	0	0	0	0	0	0	0	0
**Zotu37_*Streptococcus* species**	*Streptococcus gordonii*	104	0	9	11	39	0	7	3	25	195	14	8
**Zotu44_*Streptococcus species***	*Streptococcus sanguinis*	52	6	4	2	1	7	1	4	3	16	11	10
**Zotu46_*Streptococcus intermedius***	n/a	1	13	60	88	36	6	93	32	57	77	3	0
**Zotu59_*Streptococcus oralis_subsp._tigurinus_clade*_071**	n/a	0	0	0	1	0	0	0	0	0	0	0	0
**Zotu64_*Streptococcus* species**	*Streptococcus parasanguinis*	0	1	0	31	0	0	0	0	3	6	0	0
**Zotu93_*Streptococcus anginosus***	n/a	0	0	21	44	1	1	0	0	2	1	0	0

**Zotu97_*Streptococcus constellatus***	n/a	0	1	0	0	7	5	0	0	0	0	0	1
**Zotu133_*Streptococcus* species**	*Streptococcus cristatus*	8	0	1	2	0	0	0	0	0	0	1	2
**Zotu168_*Streptococcus anginosus***	n/a	0	0	0	1	1	0	0	0	1	1	0	0
**Zotu180_*Streptococcus* species**	*Streptococcus* species	1	0	0	0	0	0	0	0	0	0	0	0
**Zotu201_*Streptococcus* species**	*Streptococcus peroris/lactarius*	0	0	0	10	1	0	0	0	0	0	0	0
**Zotu267_*Streptococcus* species**	*Streptococcus cristatus*	0	0	0	0	0	0	0	0	0	0	0	1
**Zotu299_*Streptococcus oralis_subsp._tigurinus_clade_071***	n/a	10	0	1	5	0	3	0	0	0	0	2	3
**Zotu335_*Streptococcus* species**	*Streptococcus anginosus*	0	0	2	2	0	0	0	0	11	4	0	0
**Zotu340_*Streptococcus oralis_subsp._tigurinus_clade_071***	n/a	0	0	0	3	0	0	0	0	0	0	0	0
**Zotu341_*Streptococcus* species**	*Streptococcus* species oral taxon 056	0	0	0	3	0	1	0	0	15	10	0	0
**Zotu355_*Streptococcus* species**	*Streptococcus salivarius*	0	0	1	0	0	0	0	0	6	3	0	0

**Zotu517_*Streptococcus anginosus***	n/a	0	0	0	0	0	0	0	0	0	0	0	0
**Zotu615_*Streptococcus* species**	*Streptococcus* species	0	0	0	0	0	0	0	0	0	0	0	0
**Zotu668_*Streptococcus sobrinus***	n/a	0	0	0	0	0	0	0	0	0	0	0	0
**Zotu774_*Streptococcus parasanguinis_clade*_411**	n/a	0	0	0	2	0	0	0	0	1	0	0	0
**Zotu779_*Streptococcus* species**	*Streptococcus oralis*	0	0	0	0	0	0	0	0	0	0	0	0
**Zotu820_*Streptococcus* species**	*Streptococcus anginosus* group	0	0	0	0	0	0	0	0	0	0	0	0
**Zotu882_*Streptococcus* species**	*Streptococcus* species	0	0	0	1	0	0	0	0	0	0	0	0
**Zotu897_*Streptococcus* species**	*Streptococcus salivarius*	0	0	0	0	0	0	0	0	0	0	0	0
**Zotu933_*Streptococcus* species**	*Streptococcus* species	0	0	1	0	0	0	0	0	1	0	0	0
**Zotu1039_*Streptococcus* species**	*Streptococcus cristatus*	0	0	1	1	0	0	0	0	7	2	0	0
**Zotu1085_*Streptococcus* species**	*Streptococcus thermophilus*	0	0	0	0	0	0	0	0	2	0	0	0
**Zotu1130_*Streptococcus* species**	*Streptococcus pneumoniae*	0	0	0	0	0	0	0	0	0	0	0	0

**Zotu1174_*Streptococcus* species**	*Streptococcus anginosus*	0	0	0	0	0	0	0	0	0	0	0	0
**Zotu1200_*Streptococcus* species**	*Streptococcus gordonii*	0	0	0	1	0	0	0	0	5	5	0	0
**Zotu1214_*Streptococcus mutans***	n/a	0	0	0	38	0	0	0	0	0	0	0	0
**Zotu1218_*Streptococcus* species**	*Streptococcus* species	0	0	0	0	0	0	0	0	0	0	0	0
**Zotu1243_*Streptococcus* species**	*Streptococcus cristatus*	0	0	0	1	0	2	0	0	2	4	1	0
**Zotu1252_*Streptococcus mutans***	n/a	0	0	0	5	0	0	0	0	0	0	0	0
**Zotu1290_*Streptococcus* species**	*Streptococcus* species	0	0	0	2	0	0	0	0	0	0	0	0
**Zotu1300_*Streptococcus parasanguinis_clade_*411**	n/a	0	0	0	0	0	0	0	0	0	0	0	0
**Zotu1309_*Streptococcus* species**	no match/low cover	0	0	0	0	0	0	0	0	0	0	0	0
**Zotu1329_*Streptococcus* species**	*Streptococcus* species	0	0	0	0	0	0	0	0	0	0	0	0
**Zotu1346_*Streptococcus* species**	*Streptococcus oralis*	0	0	0	1	0	0	1	1	0	0	0	0
**Zotu1348_*Streptococcus* species**	no match/low cover	0	0	0	3	0	0	0	0	0	0	0	0
**Zotu1412_*Streptococcus* species**	no match/low cover	11	0	0	0	0	0	0	0	0	0	0	0

**Zotu1415_*Streptococcus* species**	*Streptococcus* species	0	0	0	2	0	0	0	0	0	0	0	0
**Zotu1427_*Streptococcus* species**	no match/low cover	0	0	0	2	0	0	0	0	0	0	1	0
**Zotu1430_*Streptococcus* species**	*Streptococcus parasanguinis*	0	0	0	0	0	0	0	0	0	0	0	0
**Zotu1431_*Streptococcus* species**	*Streptococcus cristatus*	0	0	0	2	0	0	0	0	0	0	0	1
**Zotu1444_*Streptococcus* species**	*Streptococcus gordonii*	0	0	0	13	0	0	0	0	0	0	0	0
**Zotu1466_*Streptococcus* species**	*Streptococcus gordonii*	0	0	0	0	0	0	0	0	1	0	0	0

HOMD, Human Oral Microbiome Database (v15.1); Zotu, zero-radius OTU

[1]BLAST classification based on ≥ 98% sequence similarity

[2]a, pre-intervention; b, post-intervention

(c) *Streptococcus* ZOTUs in saliva samples from probiotic cohort

ZOTU classification (HOMD v15.1)	BLAST[1]	2a[2]	2b	4a	4b	6a	6b	8a	8b	10a	10 b	12a	12 b	14a	14 b	16a	16b
**Zotu1_*Streptococcus salivarius***	n/a	412	379	1340	120	0	33	42	145	32	22	65	111	116	288	7	110
**Zotu2_*Streptococcus oralis_subsp._tigurinus_clade_071***	n/a	198	90	163	709	0	15	69	482	804	136	1	664	417	149	200	275
**Zotu22_*Streptococcus parasanguinis_clade_411***	n/a	11	25	17	7	0	0	86	275	27	41	1512	177	21	7	227	55
**Zotu26_*Streptococcus mutans***	n/a	5	7	0	0	0	0	0	0	0	0	0	26	207	161	0	1
**Zotu35_*Streptococcus salivarius***	n/a	0	0	0	0	0	0	0	0	0	0	0	1	0	0	0	0
**Zotu37_*Streptococcus* species**	*Streptococcus gordonii*	17	13	18	36	0	2	1	78	81	76	1	104	271	49	52	42
**Zotu44_*Streptococcus species***	*Streptococcus sanguinis*	212	103	4	3	0	0	0	166	65	223	4	14	160	127	5	3
**Zotu46_*Streptococcus intermedius***	n/a	132	125	152	76	7	106	2	9	4	10	0	10	80	11	8	11
**Zotu59_*Streptococcus oralis_subsp._tigurinus_clade_071***	n/a	0	0	2	29	0	1	809	36	64	258	67	37	35	5	145	139

**Zotu64_*Streptococcus* species**	*Streptococcus parasanguinis*	10	6	36	62	0	10	55	142	45	37	63	71	225	15	259	70
**Zotu93_*Streptococcus anginosus***	n/a	0	1	0	0	0	21	0	0	0	1	0	0	3	0	1	0
**Zotu97_*Streptococcus constellatus***	n/a	30	9	2	3	31	54	0	3	1	0	7	2	45	19	5	6
**Zotu133_*Streptococcus* species**	*Streptococcus cristatus*	21	11	9	27	0	0	0	92	55	29	0	45	47	12	28	19
**Zotu168_*Streptococcus anginosus***	n/a	0	0	0	0	0	6	0	0	0	0	0	0	2	0	0	0
**Zotu180_*Streptococcus* species**	*Streptococcus* species	0	0	0	0	0	0	3	3	1	5	2	3	2	1	6	3
**Zotu201_*Streptococcus* species**	*Streptococcus peroris/lactarius*	2	1	3	2	0	0	53	62	11	26	81	49	2	2	61	32
**Zotu267_*Streptococcus* species**	*Streptococcus cristatus*	2	1	0	0	0	0	0	6	0	8	0	0	6	7	0	0
**Zotu299_*Streptococcus oralis_subsp._tigurinus_clade_071***	n/a	28	15	36	22	0	0	2	103	37	30	3	63	48	24	19	25
**Zotu335_*Streptococcus* species**	*Streptococcus anginosus*	13	16	101	6	0	2	0	2	0	0	0	2	2	2	0	2
**Zotu340_*Streptococcus oralis_subsp._tigurinus_clade_071***	n/a	12	7	27	27	0	0	0	20	5	4	2	16	11	10	1	22

**Zotu341_*Streptococcus* species**	*Streptococcus* species oral taxon 056	26	41	87	10	0	0	1	25	9	10	19	12	22	23	2	13
**Zotu355_*Streptococcus* species**	*Streptococcus salivarius*	13	23	80	4	0	6	0	2	0	1	0	3	1	3	1	2
**Zotu517_*Streptococcus anginosus***	n/a	0	0	0	0	0	0	0	0	0	0	0	0	0	0	0	0
**Zotu615_*Streptococcus* species**	*Streptococcus* species	9	3	47	10	0	1	2	17	4	0	0	13	5	21	5	16
**Zotu668_*Streptococcus sobrinus***	n/a	37	8	0	0	0	0	0	0	0	0	0	0	0	0	0	0
**Zotu774_*Streptococcus parasanguinis_clade_*411**	n/a	1	0	5	0	0	0	2	2	0	1	5	3	4	0	0	1
**Zotu779_*Streptococcus* species**	*Streptococcus oralis*	0	1	12	7	0	0	0	7	3	1	0	6	1	3	0	13
**Zotu820_*Streptococcus* species**	*Streptococcus anginosus* group	0	0	4	0	0	0	0	0	0	0	0	0	0	0	0	0
**Zotu882_*Streptococcus* species**	*Streptococcus* species	3	6	0	0	0	0	0	3	0	1	0	0	2	2	0	0
**Zotu897_*Streptococcus* species**	*Streptococcus salivarius*	2	1	7	3	0	0	1	1	0	0	0	3	3	3	0	3
**Zotu933_*Streptococcus* species**	*Streptococcus* species	0	1	3	0	0	0	0	7	0	3	1	4	2	2	6	2
**Zotu1039_*Streptococcus* species**	*Streptococcus cristatus*	15	21	118	41	0	1	8	45	10	2	0	33	18	38	2	42

**Zotu1085_*Streptococcus* species**	*Streptococcus thermophilus*	15	17	24	1	0	0	0	18	1	3	4	1	11	18	0	4
**Zotu1130_*Streptococcus* species**	*Streptococcus pneumoniae*	0	0	0	12	0	0	0	0	0	0	0	1	2	0	2	2
**Zotu1174_*Streptococcus* species**	*Streptococcus anginosus*	0	0	3	2	0	0	0	0	0	0	0	0	0	0	0	0
**Zotu1200_*Streptococcus* species**	*Streptococcus gordonii*	0	1	0	1	0	0	0	0	0	0	0	1	8	8	0	0
**Zotu1214_*Streptococcus mutans***	n/a	0	1	0	0	0	0	0	0	0	0	0	12	29	17	0	0
**Zotu1218_*Streptococcus* species**	*Streptococcus* species	0	0	2	0	0	0	0	0	0	0	0	0	0	0	0	0
**Zotu1243_*Streptococcus* species**	*Streptococcus cristatus*	2	6	18	18	0	3	0	4	0	0	0	3	18	2	4	3
**Zotu1252_*Streptococcus mutans***	n/a	0	2	0	0	0	0	0	0	0	0	0	1	6	21	0	0
**Zotu1290_*Streptococcus* species**	*Streptococcus* species	0	0	0	0	0	0	0	0	0	0	0	0	7	6	0	0
**Zotu1300_*Streptococcus parasanguinis_clade*_411**	n/a	0	1	4	2	0	0	2	5	0	0	1	1	2	1	1	6
**Zotu1309_*Streptococcus* species**	no match/low cover	0	2	0	1	0	0	0	13	0	2	9	17	1	0	7	1
**Zotu1329_*Streptococcus* species**	*Streptococcus* species	0	0	2	0	0	0	1	4	1	0	2	1	1	0	0	1

**Zotu1346_*Streptococcus* species**	*Streptococcus oralis*	3	1	5	11	0	3	1	1	1	2	0	2	6	0	0	0
**Zotu1348_*Streptococcus* species**	no match/low cover	2	2	0	3	0	0	0	4	1	0	0	6	6	1	1	0
**Zotu1412_*Streptococcus* species**	no match/low cover	0	0	0	0	0	0	0	0	0	0	1	0	0	0	0	0
**Zotu1415_*Streptococcus* species**	*Streptococcus* species	0	0	0	0	0	0	0	0	0	0	0	0	3	2	0	0
**Zotu1427_*Streptococcus* species**	no match/low cover	0	0	0	0	0	0	0	0	0	1	0	1	0	0	0	0
**Zotu1430_*Streptococcus* species**	*Streptococcus parasanguinis*	0	1	0	1	0	0	6	3	0	1	1	0	1	0	0	3
**Zotu1431_*Streptococcus* species**	*Streptococcus cristatus*	0	0	0	0	0	3	0	0	0	0	0	0	6	0	1	0
**Zotu1444_*Streptococcus* species**	*Streptococcus gordonii*	0	0	0	0	0	0	0	0	0	0	0	0	1	0	0	0
**Zotu1466_*Streptococcus* species**	*Streptococcus gordonii*	0	0	0	1	0	0	0	0	0	0	0	0	0	0	0	0

HOMD, Human Oral Microbiome Database (v15.1); Zotu, zero-radius OTU

[1]BLAST classification based on ≥ 98% sequence similarity

[2]a, pre-intervention; b, post-intervention

(d) *Streptococcus* ZOTUs in saliva samples from placebo cohort

ZOTU classification (HOMD v15.1)	BLAST[1]	3a[2]	3b	7a	7b	9a	9b	13a	13b	15a	15b	17a	17b
**Zotu1_*Streptococcus salivarius***	n/a	0	428	204	907	8	268	262	330	213	264	48	36
**Zotu2_*Streptococcus oralis_subsp._tigurinus_clade_*071**	n/a	109	206	238	232	505	221	346	338	143	43	136	122
**Zotu22_*Streptococcus parasanguinis_clade_*411**	n/a	11	46	90	132	514	118	204	276	76	220	22	10
**Zotu26_*Streptococcus mutans***	n/a	0	0	0	229	0	0	7	13	0	1	0	1
**Zotu35_*Streptococcus salivarius***	n/a	0	0	0	0	0	0	0	0	0	0	0	0
**Zotu37_*Streptococcus* species**	*Streptococcus gordonii*	86	45	37	23	90	53	51	45	289	240	16	11
**Zotu44_*Streptococcus species***	*Streptococcus sanguinis*	580	6	76	54	28	38	11	23	45	65	11	5
**Zotu46_*Streptococcus intermedius***	n/a	7	6	117	79	6	2	13	6	31	16	0	1
**Zotu59_*Streptococcus oralis_subsp._tigurinus_clade_*071**	n/a	6	98	0	6	45	14	9	21	4	8	24	7
**Zotu64_*Streptococcus* species**	*Streptococcus parasanguinis*	36	128	5	29	144	59	45	170	57	183	29	15

**Zotu93_*Streptococcus anginosus***	n/a	0	0	48	18	0	0	0	0	0	0	0	0
**Zotu97_*Streptococcus constellatus***	n/a	5	5	0	0	69	39	0	0	0	0	3	2
**Zotu133_*Streptococcus* species**	*Streptococcus cristatus*	31	22	10	17	67	33	29	36	15	12	13	2
**Zotu168_*Streptococcus anginosus***	n/a	0	0	2	1	0	0	0	0	0	0	0	0
**Zotu180_*Streptococcus* species**	*Streptococcus* species	1	1	0	0	2	2	3	1	0	5	0	0
**Zotu201_*Streptococcus* species**	*Streptococcus peroris/lactarius*	3	21	5	15	123	20	44	45	6	7	4	0
**Zotu267_*Streptococcus* species**	*Streptococcus cristatus*	7	1	0	2	0	0	0	0	0	3	0	0
**Zotu299_*Streptococcus oralis_subsp._tigurinus_clade_071***	n/a	73	16	14	20	104	28	71	47	17	8	13	8
**Zotu335_*Streptococcus* species**	*Streptococcus anginosus*	0	4	3	17	0	4	0	1	3	3	0	0
**Zotu340_*Streptococcus oralis_subsp._tigurinus_clade_071***	n/a	3	27	2	20	11	8	30	20	8	2	2	4
**Zotu341_*Streptococcus* species**	*Streptococcus* species oral taxon 056	0	33	0	32	6	30	23	40	59	94	5	0

**Zotu355_*Streptococcus* species**	*Streptococcus salivarius*	0	5	1	17	0	8	2	0	5	2	0	0
**Zotu517_*Streptococcus anginosus***	n/a	0	0	1	0	0	0	0	0	0	0	0	0
**Zotu615_*Streptococcus* species**	*Streptococcus* species	0	28	1	22	7	11	24	31	10	8	5	1
**Zotu668_*Streptococcus sobrinus***	n/a	0	0	0	0	0	0	0	0	0	0	0	0
**Zotu774_*Streptococcus parasanguinis_clade*_411**	n/a	0	5	0	8	0	4	4	6	4	15	1	0
**Zotu779_*Streptococcus* species**	*Streptococcus oralis*	0	14	0	5	0	6	9	13	3	1	3	1
**Zotu820_*Streptococcus* species**	*Streptococcus anginosus* group	0	0	0	0	0	0	0	0	0	0	0	0
**Zotu882_*Streptococcus* species**	*Streptococcus* species	0	0	0	0	0	0	0	0	0	2	1	0
**Zotu897_*Streptococcus* species**	*Streptococcus salivarius*	0	4	1	3	0	5	1	4	2	2	1	0
**Zotu933_*Streptococcus* species**	*Streptococcus* species	2	3	1	3	12	3	2	6	0	1	1	1
**Zotu1039_*Streptococcus* species**	*Streptococcus cristatus*	0	65	9	47	2	44	50	57	53	49	9	6
**Zotu1085_*Streptococcus* species**	*Streptococcus thermophilus*	0	6	3	8	1	12	13	12	11	25	2	1

**Zotu1130_*Streptococcus* species**	*Streptococcus pneumoniae*	0	0	0	0	2	2	0	0	0	0	0	0
**Zotu1174_*Streptococcus* species**	*Streptococcus anginosus*	0	0	0	0	0	0	0	0	1	0	0	0
**Zotu1200_*Streptococcus* species**	*Streptococcus gordonii*	0	2	0	2	0	0	3	3	21	16	1	1
**Zotu1214_*Streptococcus mutans***	n/a	0	0	0	14	0	0	0	2	0	0	0	0
**Zotu1218_*Streptococcus* species**	*Streptococcus* species	0	0	0	0	0	0	0	0	0	0	0	0
**Zotu1243_*Streptococcus* species**	*Streptococcus cristatus*	1	3	4	1	21	2	3	0	1	3	1	0
**Zotu1252_*Streptococcus mutans***	n/a	0	0	0	37	0	0	0	2	0	0	1	0
**Zotu1290_*Streptococcus* species**	*Streptococcus* species	0	0	0	1	0	0	0	0	0	0	0	0
**Zotu1300_*Streptococcus parasanguinis_clade*_411**	n/a	0	24	0	0	0	4	4	7	4	10	1	0
**Zotu1309_*Streptococcus* species**	no match/low cover	5	0	0	3	4	4	11	5	16	24	0	0
**Zotu1329_*Streptococcus* species**	*Streptococcus* species	0	3	0	0	0	5	3	7	1	3	0	0
**Zotu1346_*Streptococcus* species**	*Streptococcus oralis*	0	0	0	1	0	1	2	2	0	0	0	0
**Zotu1348_*Streptococcus* species**	no match/low cover	0	0	0	3	0	2	6	2	32	21	0	0

**Zotu1412_*Streptococcus* species**	no match/low cover	54	0	0	0	0	0	0	0	0	0	0	0
**Zotu1415_*Streptococcus* species**	*Streptococcus* species	0	0	0	0	0	0	0	0	0	0	0	0
**Zotu1427_*Streptococcus* species**	no match/low cover	0	0	0	0	0	0	0	0	0	0	0	0
**Zotu1430_*Streptococcus* species**	*Streptococcus parasanguinis*	0	11	0	4	0	1	3	6	4	8	1	1
**Zotu1431_*Streptococcus* species**	*Streptococcus cristatus*	0	0	0	0	2	2	0	0	1	0	0	0
**Zotu1444_*Streptococcus* species**	*Streptococcus gordonii*	0	1	0	0	0	0	0	0	0	0	0	0
**Zotu1466_*Streptococcus* species**	*Streptococcus gordonii*	0	0	0	0	0	0	0	0	4	0	0	0

HOMD, Human Oral Microbiome Database (v15.1); Zotu, zero-radius OTU

[1]BLAST classification based on ≥ 98% sequence similarity
[2]a, pre-intervention; b, post-intervention
