# Supplementary material for: Randomised, double-blind, placebo-controlled trial of oral probiotic Streptococcus salivarius M18 on head and neck cancer patients post-radiotherapy: a pilot study
Source: Sci Rep. 2020 Aug 6;10:13201. doi: 10.1038/s41598-020-70024-y (PMC7411050; doi:10.1038/s41598-020-70024-y)
Supplement: Supplementary file 3 — Supplementary Table S3. [file 41598_2020_70024_MOESM3_ESM.pdf]

Supplementary Table S3. Interactions with ZOTU1_*Streptococcus salivarius*

(a) Plaque

ZOTU	Taxonomic classification (HOMD v15.1)/BLASTn	Inverse covariance
Zotu1039	*Streptococcus cristatus*	0.376445
Zotu50	*Atopobium parvulum*	0.31431
Zotu217	*Prevotella oris*	0.140236
Zotu782	*Capnocytophaga* spp. HMT 336	0.073003
Zotu68	*Atopobium* spp.	0.04168
Zotu744	*Prevotella oulorum*	0.039413
Zotu1085	*Streptococcus thermophilus*	0.027808
Zotu145	*Prevotella oris*	0.005125
Zotu1380	*Alloprevotella rava*	0.000449
Zotu589	*Selenomonas* spp.	-0.00357
Zotu1037	*Treponema maltophilum*	-0.01185
Zotu1366	*Prevotella marshii*	-0.01484
Zotu90	*Treponema socranskii*	-0.02036
Zotu265	*Atopobium* spp.	-0.02814
Zotu238	*Fretibacterium* spp.	-0.0299
Zotu160	*Campylobacter rectus*	-0.03213
Zotu211	*Ruminococcaceae* [G1] HMT 075	-0.03419
Zotu253	*Leptotrichia* spp. HMT 223	-0.0366
Zotu611	*Selenomonas* spp.	-0.03835
Zotu2	*Streptococcus oralis* subsp. *tigurinus*	-0.04169
Zotu530	*Prevotella* spp. HMT 301	-0.09091
Zotu586	*Peptococcus* spp. HMT 167	-0.10722

Zotu, zero-radius OTU; HOMD, Human Oral Microbiome Database (v15.1); HMT, human microbial taxon

(b) Saliva

ZOTU	Taxonomic classification (HOMD v15.1)/BLASTn	Inverse covariance
Zotu1039	*Streptococcus cristatus*	0.308078
Zotu897	*Streptococcus salivarius*	0.306017
Zotu1085	*Streptococcus thermophilus*	0.19516
Zotu341	*Streptococcus* spp. oral taxon 056	0.146117
Zotu63	*Veillonella parvula*	0.050931
Zotu335	*Streptococcus anginosus* group	0.044688
Zotu344	*Selenomonas artemidis*	0.039257
Zotu83	*Prevotella* spp.	0.03785
Zotu1412*	No match/low cover	-0.01215
Zotu384	*Prevotella* spp. HMT 526	-0.01419
Zotu349	*Lachnospiraceae* [G8] HMT 500	-0.01599
Zotu79	*Fusobacterium nucleatum* subsp. *vincentii*	-0.09284
Zotu103	*Leptotrichia* spp.	-0.12163

Zotu, zero-radius OTU; HOMD, Human Oral Microbiome Database (v15.1); HMT, human microbial taxon

* Not included in network
